# Effects of selectively assisting impaired subtasks of walking in chronic stroke survivors

**DOI:** 10.1186/s12984-020-00762-7

**Published:** 2020-10-28

**Authors:** Simone S. Fricke, Hilde J. G. Smits, Cristina Bayón, Jaap H. Buurke, Herman van der Kooij, Edwin H. F. van Asseldonk

**Affiliations:** 1 Department of Biomechanical Engineering, University of Twente, Enschede, The Netherlands; 2 Department of BioMechanical Engineering, Delft University of Technology, Delft, The Netherlands; 3 Roessingh Research and Development, Enschede, The Netherlands; 4 Department of Biomedical Signals and System, University of Twente, Enschede, The Netherlands

**Keywords:** Robotic gait training, Rehabilitation, Gait, Stroke, Assist-as-needed, Subtask-based assistance

## Abstract

**Background:**

Recently developed controllers for robot-assisted gait training allow for the adjustment of assistance for specific subtasks (i.e. specific joints and intervals of the gait cycle that are related to common impairments after stroke). However, not much is known about possible interactions between subtasks and a better understanding of this can help to optimize (manual or automatic) assistance tuning in the future. In this study, we assessed the effect of separately assisting three commonly impaired subtasks after stroke: foot clearance (FC, knee flexion/extension during swing), stability during stance (SS, knee flexion/extension during stance) and weight shift (WS, lateral pelvis movement). For each of the assisted subtasks, we determined the influence on the performance of the respective subtask, and possible effects on other subtasks of walking and spatiotemporal gait parameters.

**Methods:**

The robotic assistance for the FC, SS and WS subtasks was assessed in nine mildly impaired chronic stroke survivors while walking in the LOPES II gait trainer. Seven trials were performed for each participant in a randomized order: six trials in which either 20% or 80% of assistance was provided for each of the selected subtasks, and one baseline trial where the participant did not receive subtask-specific assistance. The influence of the assistance on performances (errors compared to reference trajectories) for the assisted subtasks and other subtasks of walking as well as spatiotemporal parameters (step length, width and height, swing and stance time) was analyzed.

**Results:**

Performances for the impaired subtasks (FC, SS and WS) improved significantly when assistance was applied for the respective subtask. Although WS performance improved when assisting this subtask, participants were not shifting their weight well towards the paretic leg. On a group level, not many effects on other subtasks and spatiotemporal parameters were found. Still, performance for the leading limb angle subtask improved significantly resulting in a larger step length when applying FC assistance.

**Conclusion:**

FC and SS assistance leads to clear improvements in performance for the respective subtask, while our WS assistance needs further improvement. As effects of the assistance were mainly confined to the assisted subtasks, tuning of FC, SS and WS can be done simultaneously. Our findings suggest that there may be no need for specific, time-intensive tuning protocols (e.g. tuning subtasks after each other) in mildly impaired stroke survivors.

## Background

Robot-assisted gait training (RAGT) has been developed to improve therapy after neurological disorders (e.g. stroke) by providing intensive and task-specific training while decreasing physical load for therapists. The use of robotic devices can positively affect gait training after stroke, especially when combined with common physical therapy and in the most impaired patients in the (sub-)acute phase after stroke [1–3].

Previous studies suggest that RAGT can be further improved by personalizing training and promoting active participation since active participation is an essential factor in gait recovery and motor learning after stroke [4–7]. To improve active participation, various controllers, based on the assist-as-needed principle (AAN, i.e. only assisting the patient when needed), have been developed [8–11]. Some of these current AAN controllers either set a specific assistance level for the whole gait cycle, or they adjust the assistance for each instance of the gait cycle (e.g. each percentage) [7, 9, 12]. Others focus on assisting specific joints and intervals of the gait cycle that are related to impairments after stroke (also called subtasks) [10, 11, 13, 14]. The assistance is changed for these subtasks based on deviations from reference trajectories [11, 13]. Assistance is applied for the (most) impaired subtasks, while subtask-based assistance allows the user to move freely during other, non-assisted, portions of the gait cycle.

A better understanding about the exact effect of subtask-based assistance on gait is needed to help with manual assistance tuning (i.e. tuning done by therapists), and optimize controllers that automatically tune assistance during RAGT. Recently, we developed an automatically-tuned subtask-based controller and tested it in people with stroke and spinal cord injury [13]. This controller simultaneously adjusted the assistance for various subtasks of gait. However, whether interactions between subtasks affect this assistance tuning process is not known. For example, if the performance on one subtasks is limiting the overall gait performance, assistance on this subtask could lead to a widespread improvement on various other subtasks. In this case, only assistance on this ‘bottleneck’ subtask would be needed and not on each of the separate subtasks. We do not yet know whether these interactions occur and how they should be incorporated in the control.

In this study, we assessed the effect of assistance during walking for three of the most common impairments after stroke: (1) insufficient knee flexion during swing phase (foot clearance (FC) subtask), (2) increased knee flexion or hyperextension during stance phase (stability during stance (SS) subtask) and (3) problems with shifting the weight towards the paretic leg (weight shift (WS) subtask) [15, 16]. For each of these subtasks, only little is known about the effect on other intervals of the gait cycle and spatiotemporal parameters in stroke survivors:

*Foot clearance (FC):* Previous experiments in stroke survivors receiving foot clearance assistance in robotic gait trainers or using powered orthoses have also shown some effects, although minor, on other parts of the gait cycle. For example, foot clearance assistance in the LOPES I and LOPES II gait trainer resulted in an increase in knee and hip angles during the swing phase and a larger step height [12, 17]. No significant effects on other spatiotemporal gait parameters were found in these studies. In addition, Sulzer et al. [18] found a small increase in peak hip abduction (2°) when assisting knee flexion during the pre-swing phase with a powered knee orthosis (Series Elastic Remote Knee Actuator, SERKA).

*Stability during stance (SS):* To the best of our knowledge, so far no study investigated the effect of robotic assistance during the stance phase in stroke survivors [19]. In children with cerebral palsy, knee extension assistance did not lead to significant changes in step length and step width, however, an increase in peak stance knee and hip extension was found [20]. This shows the potential of SS assistance to also improve other aspects of walking.

*Weight shift (WS):* Only little is known about the effect of weight shift assistance. Next to improving lateral pelvis movement, weight shift assistance improved step length symmetry in a stroke survivor in LOPES II [10]. After incomplete spinal cord injury (injury level between C4 and T10), weight shift assistance with another robotic device also led to an increased step length in the weaker leg of the participants [21].

To sum up, sometimes small effects of assisting FC, SS and WS were found for specific subtasks in small groups of people with neurological disorders (1–9 participants per study). However, in most previous studies only a limited number of outcome measures were analyzed. The goal of the current study was to determine how robotic assistance for FC, SS and WS subtasks influences the gait pattern within one session. We analyzed the performance for various other subtasks and spatiotemporal gait parameters (e.g. step length, stance time). We expected that assistance for a specific subtask clearly improves performance for the assisted subtask, but could also influence other portions of the gait pattern. Findings from this study can lead to improvements in robotic gait training by optimizing assistance tuning and targeting assistance better towards the specific needs of the patient.

## Methods

### Participants

Nine chronic stroke survivors (> 6 months after stroke) participated in this study (7 male, 56 ± 12 years, height 1.78 ± 0.06 m, weight 83.4 ± 8.9 kg). Information about the participants, their clinical scores and settings while walking in LOPES II can be found in Table 1.

**Table 1:** Overview of participants’ characteristics, clinical scores and settings for LOPES II

ID	Gender	Age	Time post stroke (months)	Paretic leg	10MWT (km/h)	Assistive device(s) used during 10MWT	FAC (max. 5)	FMA (max. 34)	MI (max. 99)	Walking speed in LOPES II (km/h)	Toe-lifter in LOPES II
P1	m	55	57	r	2.5	–	5	15	28	1.2	Yes
P2	m	61	28	l	2.4	cane, AFO	5	20	53	1.5	Yes
P3	m	74	55	r	4.0	–	5	28	64	1.5	No
P4	m	54	25	r	1.9	cane, AFO	5	11	28	1.2	Yes
P5	m	52	15	r	2.1	cane	5	14	50	0.8	Yes
P6	m	77	28	r	3.6	AFO	5	30	53	1.5	No
P7	m	55	36	l	4.2	–	5	31	83	1.6	No
P8	f	33	133	l	3.6	–	5	21	61	2.0	No
P9	f	56	47	r	3.5	SCO	5	27	83	1.6	Yes

ID: identification code used for each specific participant, 10MWT: 10 meter walking test, FAC: functional ambulation category, FMA: Fugl-Meyer assessment (lower extremity), MI: Motricity index (lower extremity), AFO: ankle foot orthosis, SCO: stance control orthosis. Maximal clinical scores are given between brackets. More information on the clinical scores and their interpretation can be found in [23–25, 30]

**Table 2:**
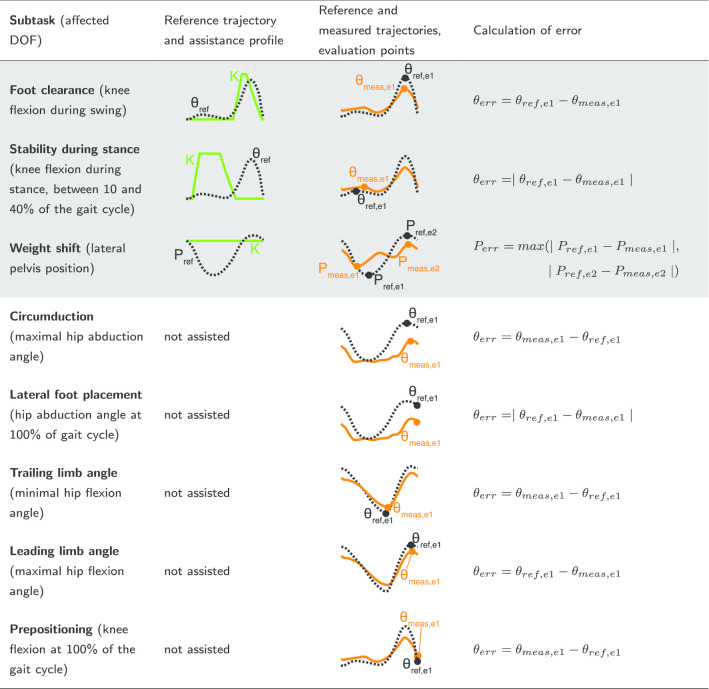
Overview of assistance and evaluated variables for different subtasks

Reference (black dotted lines) and example measured data (orange lines) positions and joint angles ($\hbox {P}_{\mathrm{ref}}$, $\hbox {P}_{\mathrm{ref}}$, $\uptheta _{\mathrm{ref}}$, $\uptheta _{\mathrm{ref}}$), assistance profiles (K) and evaluation points (e.g. $\hbox {P}_{\mathrm{ref,e1}}$) that were used to calculate the error are shown. Each figure shows one gait cycle starting with left heel strike at 0%. Weight shift to the right side, abduction and flexion angles are defined positive. The subtasks that were assisted in this study are shown with a grey background

The inclusion criteria were (1) diagnosis of a hemiparesis as a result of a stroke that occurred more than 6 months prior to the study, (2) a stable medical and physical condition that allowed for three minutes of walking, (3) an age of at least 18 years, and (4) sufficient cognitive abilities to follow instructions and report any discomfort. Exclusion criteria were severe spasms that can interfere with the functions of LOPES II.

The experimental protocol was approved by the local medical ethical committee (METC Twente, Enschede, The Netherlands) and participants gave written informed consent prior to their participation.

### Robotic gait trainer and assistance

The LOPES II robotic gait trainer was used in this study to provide robotic assistance to the participants during walking. LOPES II is a device that is attached to the user with push–pull rods at the pelvis and lower legs [10]. While the user is walking on an instrumented treadmill (custom-built treadmill, Motek Medical, Amsterdam, The Netherlands), the device can assist movements in eight degrees of freedom (DOFs): pelvis anteroposterior, pelvis mediolateral, and hip abduction/adduction, hip flexion/extension and knee flexion/extension for both legs. LOPES II is admittance controlled and the robot can be tuned from transparent mode (0% assistance, minimizing interaction forces between the device and the user) to full assistance (100% assistance, mimicking position control).

LOPES II can move the user along reference trajectories when applying assistance. For each DOF, a reference trajectory is defined based on a data set from healthy, elderly subjects [22] and previous experiences with these reference trajectories [11, 13]. The amount of assistance that is applied by LOPES II depends on deviations from these reference trajectories and virtual spring stiffnesses (with equilibrium positions on the reference trajectories). This virtual spring stiffness *K* is calculated with the following equation for each DOF (*j*) and each instant (*i* in %) of the gait cycle:

$$\begin{aligned} K_{j,i}=K_{max,j} \left( \frac{G_{j,i}}{100}\right) ^2 \end{aligned}$$

where $K_{max,j}$ is a maximal stiffness that is defined for each DOF of LOPES II and $G_{j,i}$ is the desired assistance [10].

For each DOF, a subtask-based assistance can be applied with LOPES II, i.e. assisting only specific intervals of the gait cycle for the respective DOF (e.g. for foot clearance only swing phase for the knee). In this study, we assisted the FC, SS and WS subtasks, as these subtasks are often the most impaired subtasks after stroke. The assistance profiles for these subtasks are shown in Table 2 together with other non-assisted subtasks that were used to assess the effect of assistance for FC, SS and WS. Each subtask was assisted separately at a low (20%) and a high level (80%), depending on the trial (see “Experimental procedures” section).

In addition to the subtask-based assistance, a general assistance was applied, meaning that the whole gait pattern was assisted (i.e. all DOFs during the whole gait cycle with a constant assistance level). The minimal impedance mode of LOPES II is not completely transparent and especially for the pelvis the virtual inertia is rather high (40 kg) [10]. We applied a general assistance of 3% to make walking in the device without any additional assistance easier, especially for stroke survivors.

### Experimental procedures

Each participant attended two sessions (familiarization and experimental session) on two different days. In the familiarization session, clinical assessment was performed by an experienced physical therapist. The following clinical scores were determined: Functional Ambulation Category (FAC) [23], comfortable walking speed in the 10 meter walking test (10MWT) [24], Fugl-Meyer Assessment (FMA) [25] for the lower extremity and Motricity Index (MI) [26] for the lower extremity (Table 1). After this, participants walked for three trials in LOPES II to get used to the device and to choose a comfortable walking speed. No body weight support was applied by the LOPES II system, however, participants used the hand rails of LOPES II during all trials in both sessions. If participants were not able to lift their toes/feet enough during swing phase (i.e. prematurely hitting the ground), a passive toe-lifter was used on the more impaired leg. In the first two trials of the familiarization session, participants received various amounts of general assistance (max. 30%) to get used to LOPES II. In the last trial, 3% of general assistance was applied to make sure that they were able to walk in this condition. No individual assistance (FC, SS, WS) was applied in this familiarization session.

**Fig. 1:**
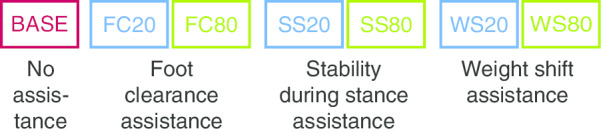
Overview of the seven trials of the experimental session. Each participant took part in all trials. The order of the seven trials was randomized and each trial took 2 minutes. BASE: baseline, WS: weight shift, FC: foot clearance, SS: stability during stance. No subtask-specific assistance was applied in BASE. 20 means 20% of assistance and 80 means 80% of assistance for the respective subtask. For all other portions of the gait cycle 3% of general assistance was provided in all trials (including BASE)

In the experimental session (Fig. 1), participants started with a familiarization trial of 3 min to get used to walking in LOPES II again, receiving 3% general assistance. After this, seven trials (2 min each), which were used for data analysis in this study, were performed (Fig. 1). The order of the trials was randomized between participants. In the baseline trial (BASE), no subtask-based assistance was applied and participants received only 3% general assistance. In the other trials, 3% general assistance was applied for the whole gait cycle, and specific subtasks (FC, SS or WS) were assisted with either 20% or 80%.

### Outcome measures

For each trial, we calculated the performance for various subtasks of gait and spatiotemporal parameters. As explained in detail in the subsections below, these assessments were based on joint angles and joint positions measured by the LOPES II, all sampled at 1000 Hz [10]. Additionally, we performed statistics comparing the different trials (no, 20, 80% assistance) for each assisted subtask. For the analysis, the first five and last five steps were removed and not taken into account during further analyses.

#### Subtask performance

To evaluate gait performance: FC, SS, WS, circumduction, lateral foot placement, trailing and leading limb angle, and prepositioning were used (Table 2). Subtask-based performances were calculated based on deviations from reference trajectories for specific evaluations points (column 3 and 4 of Table 2).

For some subtasks, the absolute error was calculated as deviations in both directions can be found in stroke survivors. For example, for the SS subtask, some stroke survivors might have too much knee flexion during stance while others can get hyperextension of the knee. For other subtasks, we did not take the absolute error since we assumed that deviations in only one direction would be detrimental (Table 2): for example, a larger knee flexion during swing (foot clearance subtask) is acceptable. However, not enough knee flexion might lead to the toes being dragged along the ground which is detrimental for the walking pattern.

#### Spatiotemporal parameters

The following spatiotemporal parameters were assessed: step length (anterior–posterior distance between trailing and leading limb ankle at heel strike), step height (ankle height at the moment that the ankle had the same anterior–posterior position as the hip), step width (mediolateral distance between trailing and leading limb ankle at heel strike), and duration of the stance and swing phases.

#### Statistics

Friedman tests were used for the statistical analyses, to determine the effect of the assistance for a specific subtask on the performance of this specific subtask and other subtasks, and spatiotemporal parameters. The Friedman test is a non-parametric alternative to repeated measures ANOVA and does not rely on the assumption that data is normally distributed. The Friedman tests uses ranks of the observations instead of using the exact magnitudes. For example, for the FC assistance, for each subtask (including FC) and each spatiotemporal parameter, a separate Friedman test was performed to evaluate the effect of FC assistance. These tests were also performed for the other two assisted subtasks. A p-value smaller than 0.05 was considered statistically significant.

If statistically significant effects of the assistance were found, post-hoc comparisons were performed by applying the Wilcoxon signed-rank test. A Bonferroni correction was used to account for multiple comparisons which means that p-values smaller than 0.017 (0.05 divided by 3 comparisons) were considered statistically significant for the post-hoc tests.

## Results

### Effect on subtask performance and spatiotemporal parameters

#### Foot clearance (FC)

**Fig. 2:**
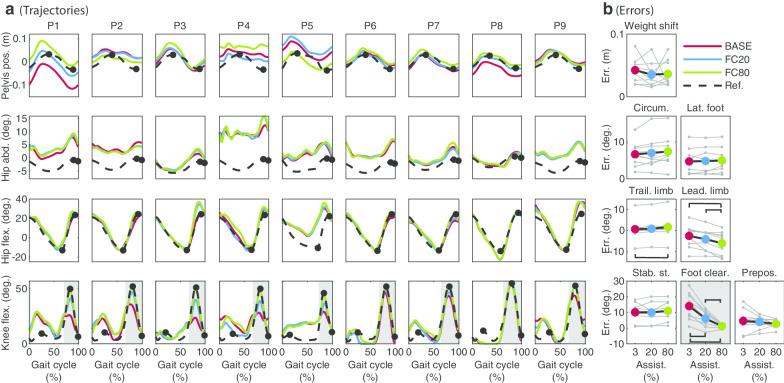
Joint trajectories (**a**) and errors (**b**) for each subtask for all participants Trajectories are shown for each individual separately. For the pelvis position, 0 is the center of the treadmill and a positive value means a deviation from the center towards the paretic side. Abduction and flexion angles are positive. The gait cycle starts with heel strike of the paretic leg. The grey area indicates the interval of the gait cycle within which the assistance was applied (**a**) and the assisted subtask (**b**). The black markers in A indicate the evaluation points. For the weight shift, the largest error of the errors for both evaluation points is used to evaluate performance (see also Table 2). For the other DOFs the first marker corresponds to the first plot with errors, the second marker to the second plot with errors etc. Each grey marker and connecting grey line in **b** show the results for one individual. The colored dots and connecting black lines show the average errors across individuals. Each black bar indicates significant differences between two conditions ($\upalpha =0.05$)

Errors for the FC subtask significantly decreased when assistance was applied (Fig. 2, ($\upchi ^2$ = 16.22, p = 0.0003)). The average error decreased from 14.1° in BASE to 6.2°. in FC20 and 1.3° in FC80. Post-hoc comparisons show significant differences for BASE-FC20, BASE-FC80 (p = 0.0039 in both cases) and FC20-FC80 (p = 0.0117).

FC assistance also significantly affected the leading limb and trailing limb angle subtasks, showing consistent changes in various participants. The error for the leading limb angle subtask significantly decreased (i.e. hip flexion increased) when applying assistance ($\upchi ^2$ = 12.67, p = 0.0018, post-hoc: significant differences for BASE-FC80 (p = 0.0039) and FC20-FC80 (p = 0.0078). However, it should be considered that in eight of the nine participants the error was already close to 0°. or lower in BASE, and improvements for the leading limb angle subtask were not necessarily needed for these participants. For the trailing limb angle, significant differences were found ($\upchi ^2$ = 8.67, p = 0.0131). Although post-hoc analyses showed a significant increase of the error between BASE and FC80 (p = 0.0039), the effect was only small (about 1°).

For the rest of the analysed subtasks, no significant effect on performances was found on a group level. Still, sometimes changes for other subtasks were found in specific participants (e.g. prepositioning for P4 and P9 in Fig. 2).

**Fig. 3:**
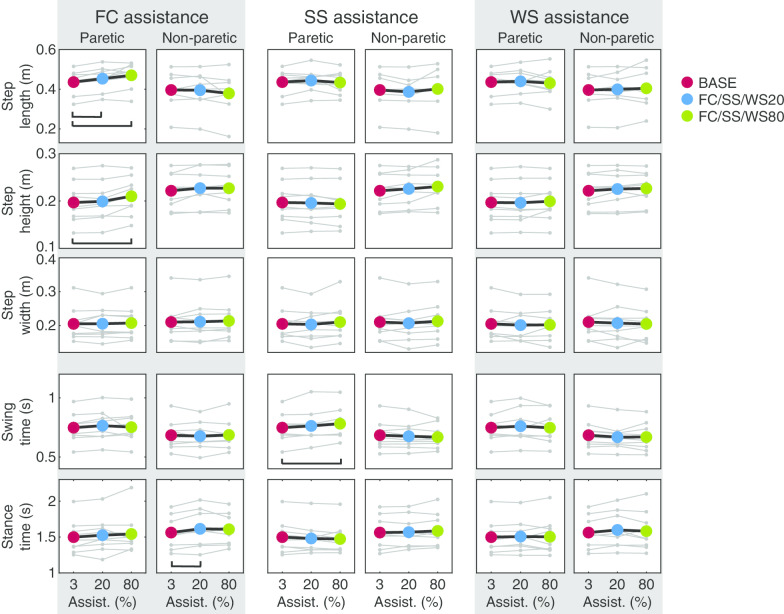
Effect of FC, SS and WS assistance on spatial (top three rows) and temporal (bottom two rows) parameters. Each marker type and connecting grey line shows the results for one individual. The colored dots and connecting black lines show the average across participants for the respective parameter. Each black bar indicates significant differences between two conditions ($\upalpha$ = 0.05)

Regarding the spatiotemporal parameters, significant effects on stance time, step length and step height of the paretic leg were found for FC assistance (Fig. 3; stance time: $\upchi ^2$ = 6.22, p = 0.0446, step length: $\upchi ^2$ = 12.29, p = 0.0021, step height: $\upchi ^2$ = 7.14, p = 0.0281). For stance time, post-hoc tests did not show significant differences between the conditions. Paretic step length was significantly increased by 0.02 m and 0.03 m for BASE-FC20 and BASE-FC80, respectively (in both cases p = 0.0117). For step height, a significant increase of 0.01 m between BASE-FC80 was found (p = 0.0117).

In addition, a change in spatiotemporal parameters was observed for the (non-assisted) non-paretic leg: stance time was significantly affected ($\upchi ^2$ = 6.22, p = 0.0446). A significant increase in stance time from 1.56 s in BASE to 1.62 s in FC20 was found (p = 0.0117). Post-hoc tests did not show any other significant differences for the pairwise comparisons.

#### Stability during stance (SS)

The errors for the SS subtask significantly decreased by applying SS assistance ($\upchi ^2$ = 18, p = 0.0001, Fig. 7). Post-hoc tests showed significant differences between BASE-SS20, BASE-SS80 and SS20-SS80 (p = 0.0039 in all cases). For none of the other subtasks a clear and significant effect of SS assistance was found on a group level.

No large changes for spatiotemporal parameters were found (Fig. 3). Only swing time of the paretic leg was significantly influenced by SS assistance ($\upchi ^2$ = 6.22, p = 0.0446). Post-hoc tests revealed a significant swing time increase of 0.03s between BASE and FC80 (p = 0.0078).

#### Weight shift (WS)

WS assistance significantly affected performance for the WS subtask ($\upchi ^2$ = 14.89, p = 0.0006). Post-hoc comparisons show a significant decrease in the error between BASE-FC20 and FC20-FC80 (in both cases p = 0.0039). Also, a significant effect was found for circumduction ($\upchi ^2$ = 6.89, p = 0.0319). However, post-hoc tests did not show significant differences between the conditions. No clear and consistent effect for all participants was found for the performance of any of the other subtasks.

**Fig. 4:**
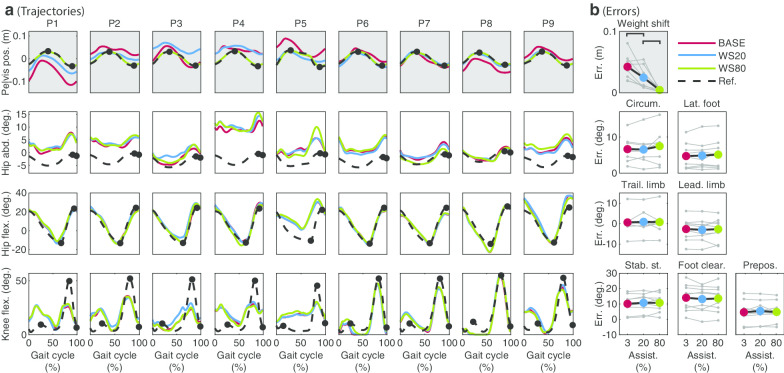
Joint trajectories (**a**) and errors (**b**) for each subtask for all participants. Trajectories are shown for each individual separately. For the pelvis position, 0 is the center of the treadmill and a positive value means a deviation from the center towards the paretic side. Abduction and flexion angles are positive. The gait cycle starts with heel strike of the paretic leg. The grey area indicates the interval of the gait cycle within which the assistance was applied (**a**) and the assisted subtask (**b**). The black markers in **a** indicate the evaluation points. For the weight shift, the largest error of the errors for both evaluation points is used to evaluate performance (see also Table 2). For the other DOFs the first marker corresponds to the first plot with errors, the second marker to the second plot with errors etc. Each grey marker and connecting grey line in **b** show the results for one individual. The colored dots and connecting black lines show the average errors across individuals. Each black bar indicates significant differences between two conditions ($\upalpha$ = 0.05)

**Fig. 5:**
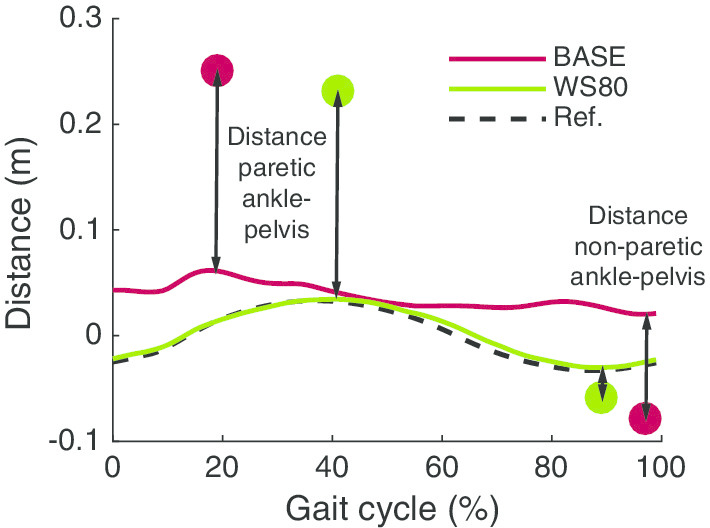
Example for the calculation of the distance between the ankles and pelvis. As an example, one step of BASE and one step of WS80 are shown. The line indicates the pelvis position and the markers indicate the position of the (paretic and non-paretic) ankle at that point of the gait cycle. A distance of 0 means exactly in the center of the treadmill, the positive axis shows deviations from the center of the treadmill towards the side of the paretic leg. For each step, the distance between the pelvis and both ankles was calculated at the time instance of maximal and minimal lateral pelvis displacement as shown

**Fig. 6:**
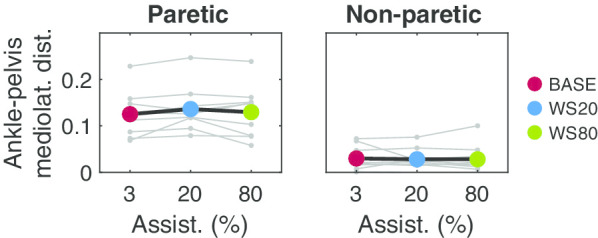
Distance between the pelvis and ankle (calculated as shown in Fig. 5) for all participants. A distance of 0 means exactly in the center of the treadmill, the positive axis shows deviations from the center of the treadmill towards the side of the respective leg. Each grey marker type shows the result for one individual. The coloured markers show the average for all included participants

The lateral pelvis movements were clearly affected by the WS assistance and all participants could closely follow the reference trajectory (Fig. 4). However, this did not necessarily imply that participants were shifting their weight more towards the paretic leg. The mediolateral distance between the paretic ankle and the pelvis was much larger than the distance between the non-paretic ankle and the pelvis (Fig. 5). Applying WS assistance moved the pelvis trajectory towards the reference trajectory, however, participants did not modify their foot placement relative to the pelvis. Consequently, for none of the participants, WS assistance resulted in changes in the distance between the ankle and pelvis for the paretic and non-paretic leg (Fig. 6).

Swing time of the non-paretic leg was significantly affected by WS assistance ($\upchi ^2$ = 6.22, p = 0.0446). Post-hoc tests did not show significant differences between specific conditions. None of the other spatiotemporal parameters were significantly affected by WS assistance.

## Discussion

The goal of this study was to determine the effect of FC, SS and WS assistance in LOPES II on the performance of the assisted subtasks, and to assess how this assistance affects the performance of other subtasks and spatiotemporal gait parameters. Performance of the assisted subtasks (FC, SS or WS) clearly improved when assistance was applied for the respective subtask. However, this selective assistance did not have much beneficial effects on the performances of other non-assisted subtasks or spatiotemporal gait parameters. Only FC assistance affected the FC subtask as well as the leading limb angle subtask resulting in a larger step length. Still, the assistance also did not have significant detrimental effects on other intervals of the gait cycle, except for a small increase in the error for the trailing limb angle for 80% FC assistance.

The lack of clear effects on other subtasks on a group level can have different causes. First, the patient population performed well for most unassisted subtasks, which means that no clear performance improvements were needed for these subtasks. However, as described below, participant-specific dependencies were found in some of the more impaired participants. Second, two of the most impaired subtasks, FC and SS, were assisted during different parts of the gait cycle (swing and stance, respectively), which makes interactions between these subtasks less likely.

### Comparison to literature

Many of our results are in agreement with previous studies that evaluated the effect of FC, SS or WS assistance. However, the comparisons should be treated carefully due to the small sample sizes of the studies (including ours, max. 9 participants).

#### Foot clearance (FC)

For FC assistance, in line with our results, Koopman et al. [12] found an increased performance for the leading limb angle subtask in healthy participants. However, the results for the effect of FC assistance on hip abduction were less consistent: Koopman et al. [12] like us, did not find any significant differences in hip abduction when assisting FC during swing. In contrast to this, Sulzer et al. [18] found an increase in hip abduction when assisting knee flexion. One of the reasons for the differences could be that Sulzer et al. applied assistance during pre-swing phase which might have influenced the gait pattern in a different way than the assistance applied later during the swing phase in our study. Besides, it should be considered that we did not find a significant change in hip abduction on a group level. However, for 7 of the 9 participants we noticed more circumduction (i.e. more hip abduction during swing) for FC80 compared to BASE (Fig. 2). Similar to results from Sulzer et al. [18], hip circumduction, which is sometimes seen as a compensatory mechanism in stiff-knee gait [15], did not decrease when applying knee flexion assistance during swing phase in our study. This also supports the results from a study by Akbas et al. [27] who claims that hip circumduction is not a compensatory mechanism for deficits in knee flexion during swing phase.

#### Stability during stance (SS)

**Fig. 7:**
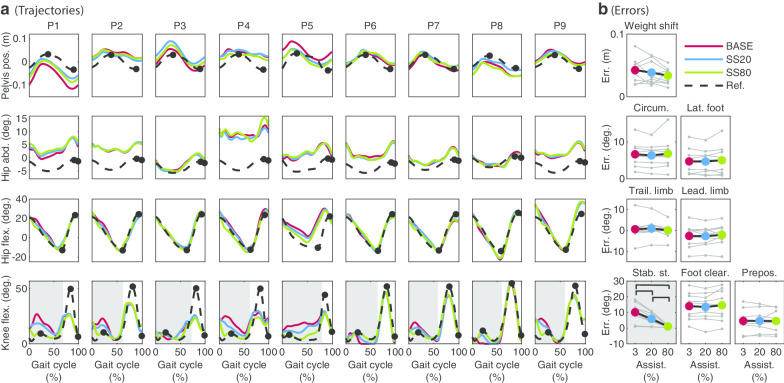
Joint trajectories (**a**) and errors (**b**) for each subtask for all participants. Trajectories are shown for each individual separately. For the pelvis position, 0 is the center of the treadmill and a positive value means a deviation from the center towards the paretic side. Abduction and flexion angles are positive. The gait cycle starts with heel strike of the paretic leg. The grey area indicates the interval of the gait cycle within which the assistance was applied (**a**) and the assisted subtask (**b**). The black markers in **a** indicate the evaluation points. For the weight shift, the largest error of the errors for both evaluation points is used to evaluate performance (see also Table 2). For the other DOFs the first marker corresponds to the first plot with errors, the second marker to the second plot with errors etc. Each grey marker and connecting grey line in **b** show the results for one individual. The colored dots and connecting black lines show the average errors across individuals. Each black bar indicates significant differences between two conditions ($\upalpha$ = 0.05)

For SS assistance, similar to a study by Lerner et al. [20] who applied assistance for the knee joint during the stance phase of walking in children with cerebral palsy, we did not find any significant effects on step length and step width. While Lerner et al. found an increase in peak hip extension during stance, in our study no clear changes in peak hip extension (trailing limb angle subtask) were found on a group level. A possible reason for this is that most of the stroke survivors already had sufficient hip extension in BASE (i.e. close to the reference trajectory) while children with crouch gait due to cerebral palsy do not have sufficient hip extension [28]. The one subject with limited hip extension in BASE in our study, did show an increase in peak hip extension resulting from SS assistance (P5 in Fig. 7).

#### Weight shift (WS)

For WS assistance, our results deviate from previous pilot experiments with two stroke survivors walking in LOPES II [17], where WS assistance resulted in a decreased hip abduction. The reason for this is that the assistance is not directly comparable: Meuleman et al. [17] applied assistance for pelvis lateral movement as well as hip abduction during the whole gait cycle. In the current study, we wanted to focus on assistance of the lateral pelvis movement only, and did not assist hip abduction.

In our study, lateral pelvic movement assistance led to smaller deviations from the pelvis reference trajectory. However, users did not adjust their foot placement relative to the pelvis position, and therefore, did not shift their weight well towards the paretic leg. Assisting hip abduction, and ensuring proper lateral foot placement relative to the pelvis [17], can be a possible solution for this problem. Another approach is to adjust the reference trajectory for the pelvis based on foot placement. This means that the reference trajectory will be defined with respect to the leading stance foot to guarantee a certain weight shift, and not with respect to the global frame (center of treadmill) as currently done.

### Study limitations

#### Patient population

When assessing the effect of assistance for selective subtasks of gait, it is crucial that participants are able to walk without (or with low levels of) assistance, while still suffering from gait impairments that can effectively be supported. The reason for this is that a high general assistance would improve performance for all subtasks, making it impossible to clearly show effects of assisting one specific subtask. Due to these requirements, only mildly impaired stroke survivors could be included. Some of these participants already followed the reference trajectories well (low errors) for the FC, SS and/or WS subtask. The good performance for most of the unassisted subtasks left only little room for improvement on these subtasks which also makes it less likely to find interactions between subtasks. For more impaired participants in which several subtasks are affected, larger interactions could occur. This is illustrated by P5 in our study, who showed improved hip extension when applying SS assistance. Despite that it is difficult to find suitable participants that fulfill the above-mentioned requirements, we recommend to extend this research to more impaired participants.

#### Handrail use

Another limitation of this study is that participants were not able to walk without using the handrails as they did not feel secure enough. A previous study showed that handrail use while walking can influence step length and step width [29]. As participants used the handrails during all trials, we expected that handrail use did not have a large influence on the differences between trials. However, performance for weight shift is likely to be influence more by handrail use. Previous (unpublished) experiments with healthy participants walking in LOPES II in minimal impedance mode have shown limited lateral pelvic movements compared to the reference trajectory when using handrails.

#### Toe-lifter

For some participants, a toe-lifter was used on the paretic leg. This toe-lifter assisted ankle dorsiflexion and prevented that the participant prematurely hit the ground during swing phase. If a participant used the toe-lifter, it was used during all trials of this participant. So, none of the observed effects of the specific subtask assistance can be attributed to the use of the toe-lifters, but they could have had a general effect on the walking pattern while walking in the LOPES.

### Future directions

Our results can be used to optimize manual and automatic assistance tuning. In tuning support levels, we strive to quickly tune the assistance levels, and apply the least amount of assistance with the largest beneficial effects to enhance active participation of the user. Our ideal case would be to find the ‘bottleneck’ subtask of walking for each individual user. Only assisting this ‘bottleneck’ subtask would result in overall improvements of gait (positive effect in several other subtasks). We did not find many clear dependencies between subtasks in mildly impaired stroke survivors. Nonetheless, our results should be taken into account by therapists while manually tuning the assistance, and for the development of automatically-tuned controllers [13]. Our findings suggest that FC, SS and WS can be tuned simultaneously without affecting each other and other non-assisted subtasks in mildly impaired stroke survivors. Based on our results, we expect that there is no need to use specific time-intensive ‘tuning protocols’ where, for example, subtasks are tuned after each other.

In people with more impairments, and possibly more interactions between subtasks (e.g. similar to P5), advantages and disadvantages of various tuning protocols should be assessed. First, subtasks could still be tuned simultaneously to reduce the time needed for assistance tuning. Second, interactions between subtasks could be taken into account. For example, similar to how therapists tune the assistance [13], we would start tuning the most impaired subtask and apply low levels of assistance for other subtasks. If more improvements are needed, other subtasks should be tuned afterwards. Although it might be difficult to find suitable participants (see “Study limitations” section), future research is required to find an optimal tuning protocol for various subtask of gait in more impaired patients.

## Conclusions

Separately assisting foot clearance and stability during stance enhances performance for the respective assisted subtask. Our weight shift assistance should be further improved in the future. Hardly any dependencies were found between the assisted subtasks and other subtasks as well as spatiotemporal parameters. Our findings suggest that FC, SS and WS can be tuned simultaneously without affecting other subtasks in mildly impaired stroke survivors. We expect that no time-intensive tuning protocols (e.g. tuning subtasks after each other) are required in these patients.

## Data Availability

The datasets generated and/or analyzed during the current study are available from the corresponding author on reasonable request.

## Abbreviations

RAGT: Robot-assisted gait therapy
LOPES: Lower extremity powered exoskeleton
DOF: Degree of freedom
10MWT: 10 meter walking test
FAC: Functional ambulation category
FMA: Fugl-Meyer assessment
MI: Motricity index
AFO: Ankle foot orthosis
SCO: Stance control orthosis

## References

[1] Mehrholz J, Thomas S, Werner C, Kugler J, Pohl M, Elsner B (2017). Electromechanical-assisted training for walking after stroke (Review). Cochr Database System Rev.

[2] Bruni MF, Melegari C, De Cola MC, Bramanti A, Bramanti P, Calabrò RS (2018). What does best evidence tell us about robotic gait rehabilitation in stroke patients: a systematic review and meta-analysis. J Clin Neurosci.

[3] Schwartz I, Meiner Z (2015). Robotic-assisted gait training in neurological patients: who may benefit?. Ann Biomed Eng.

[4] Atashzar SF, Shahbazi M, Patel RV (2019). Haptics-enabled interactive neuroRehabilitation mechatronics: classification, functionality, challenges and ongoing research. Mechatronics.

[5] Marchal-Crespo L, Reinkensmeyer DJ (2009). Review of control strategies for robotic movement training after neurologic injury. J NeuroEng Rehab.

[6] Gassert R, Dietz V (2018). Rehabilitation robots for the treatment of sensorimotor deficits: a neurophysiological perspective. J NeuroEng Rehab.

[7] Emken JL, Harkema SJ, Beres-Jones JA, Ferreira CK, Reinkensmeyer DJ (2008). Feasibility of manual teach-and-replay and continuous impedance shaping for robotic locomotor training following spinal cord injury. IEEE Trans Biomed Eng.

[8] Maggioni S, Lünenburger L, Riener R, Melendez-Calderon A (2015). Robot-Aided assessment of walking function based on an adaptive algorithm. IEEE Int Conf Rehab Robot.

[9] Maggioni S, Reinert N, Lünenburger L, Melendez-Calderon A (2018). An adaptive and hybrid end-point/joint impedance controller for lower limb exoskeletons. Front Robot AI.

[10] Meuleman J, van Asseldonk EHF, van Oort G, Rietman H, van der Kooij H (2016). LOPES II—design and evaluation of an admittance controlled gait training robot with shadow-leg approach. IEEE Trans Neural Syst Rehab Eng.

[11] Bayón C, Fricke SS, Rocon E, van der Kooij H, van Asseldonk EHF. Performance-Based adaptive assistance for diverse subtasks of walking in a robotic gait trainer: description of a new controller and preliminary results. In: Proceedings of the IEEE RAS and EMBS international conference on biomedical robotics and biomechatronics. 2018;414–419 10.1109/BIOROB.2018.8487189.

[12] Koopman B, van Asseldonk EHF, van der Kooij H (2013). Selective control of gait subtasks in robotic gait training: foot clearance support in stroke survivors with a powered exoskeleton. J NeuroEng Rehab.

[13] Fricke SS, Bayón C, van der Kooij H, van Asseldonk EHF (2020). Automatic versus manual tuning of robot-assisted Gait Training in people with neurological disorders. J NeuroEng Rehab.

[14] Chen G, Ye J, Liu Q, Duan L, Li W, Wu Z, Wang C. Adaptive control strategy for gait rehabilitation robot to assist-when-needed. In: 2018 IEEE international conference on real-time computing and robotics, RCAR. 2018. pp 538–43. 10.1109/RCAR.2018.8621706.

[15] Balaban B, Tok F (2014). Gait disturbances in patients with stroke. PM R.

[16] Olney SJ, Richards C (1996). Hemiparetic gait following stroke. Part I: characteristics. Gait Posture.

[17] Meuleman J. Design of a robot-Assisted Gait Trainer: LOPES II. Ph.D. thesis. University of Twente. 2015. 10.3990/1.9789036539654.

[18] Sulzer JS, Gordon KE, Dhaher YY, Peshkin MA, Patton JL (2010). Preswing knee flexion assistance is coupled with hip abduction in people with Stiff-Knee Gait After stroke. Stroke.

[19] Chen B, Zi B, Wang Z, Qin L, Liao WH (2019). Knee exoskeletons for gait rehabilitation and human performance augmentation: A state-of-the-art. Mech Mach Theory.

[20] Lerner ZF, Damiano DL, Bulea TC (2017). The effects of exoskeleton assisted knee extension on lower-extremity gait kinematics, kinetics, and muscle activity in children with cerebral palsy. Sci Rep.

[21] Lin J, Hsu C, Dee W, Chen D, Rymer WZ, Wu M (2019). Motor adaptation to weight shifting assistance transfers to overground walking in people with spinal cord injury. Pm&R.

[22] Koopman B, van Asseldonk EHF, van der Kooij H (2014). Speed-dependent reference joint trajectory generation for robotic gait support. J Biomech.

[23] Holden M, Gill K, Magliozzi M, Nathan J, Piehl-Baker L (1984). Clinical gait assessment in the neurologically impaired. Reliability and meaningfulness. Phys Ther.

[24] Collen FM, Wade DT, Bradshaw CM (1990). Mobility after stroke: reliability of measures of impairment and disability. Disab Rehab.

[25] Fugl-Meyer AR, Jääskö L, Leyman I, Olsson S (1975). The post-stroke hemiplegic patient. 1. a method for evaluation of physical performance. Scand J Rehab Med.

[26] Wade DT (1992). Measurement in neurological rehabilitation.

[27] Akbas T, Prajapati S, Ziemnicki D, Tamma P, Gross S, Sulzer J (2019). Hip circumduction is not a compensation for reduced knee flexion angle during gait. J Biomech.

[28] Armand S, Decoulon G, Bonnefoy-Mazure A (2016). Gait analysis in children with cerebral palsy. EFORT Open Rev.

[29] Ijmker T, Lamoth CJ, Houdijk H, Tolsma M, Van Der Woude LHV, Daffertshofer A, Beek PJ (2015). Effects of handrail hold and light touch on energetics, step parameters, and neuromuscular activity during walking after stroke. J NeuroEng Rehab.

[30] Ablity Lab: Rehabilitation measures database. 2020. https://www.sralab.org/rehabilitation-measures. Accessed 29 Aug 2020.

